# Prevalence, risk awareness and health beliefs of behavioural risk factors for cardiovascular disease among university students in nine ASEAN countries

**DOI:** 10.1186/s12889-018-5142-1

**Published:** 2018-02-13

**Authors:** Karl Peltzer, Supa Pengpid

**Affiliations:** 1 Department for Management of Science and Technology Development, Ton Duc Thang University, Ho Chi Minh City, Vietnam; 2 Faculty of Pharmacy, Ton Duc Thang University, Ho Chi Minh City, Vietnam; 3 ASEAN Institute for Health Development, Mahidol University, Salaya, Thailand; 4 Department of Research Development and Innovation, University of Limpopo, Polokwane, South Africa

**Keywords:** Overweight, Physical activity, Tobacco use, Binge drinking, Dietary behaviour, Risk awareness, Health benefits, University students, ASEAN

## Abstract

**Background:**

Understanding behavioural risk factors of cardiovascular disease (CVD) is of great importance for CVD prevention and control. The aim of the study was to investigate the prevalence, risk awareness and health beliefs of behavioural risk factors of cardiovascular disease among university students in Association of Southeast Asian Nations (ASEAN) member states.

**Methods:**

In a cross-sectional survey 8806 (37.5% male and 62.5% female) university students (Mean age 20.6, SD = 2.0) from nine ASEAN countries responded to an anonymous questionnaire.

**Results:**

Results indicate that across all nine countries, among men and women, 27.5% and 16.9%, respectively, were overweight or obese, 39.0% and 53.0% engaged in low physical activity, 6.9% and 2.5% were current tobacco users, 10.1% and 4.2% had engaged in binge drinking in the past month and 62.7% and 58.2%, respectively, did not avoid eating fat and cholesterol. After adjusting for socio-demographic factors, health status and health benefits, poor risk awareness was associated with tobacco use and binge drinking, and after adjusting for socio-demographic factors, health status and risk awareness, poorer health benefits beliefs predicted overweight, low physical activity, tobacco use, binge drinking and non-avoidance of fat and cholesterol.

**Conclusion:**

The study found a high prevalence of behavioural risk factors of CVD. Results may inform health promotion strategies among university students in ASEAN.

## Background

Cardiovascular disease (CVD) is the most prevalent cause of mortality globally [1]. At least three quarters of the world’s deaths from CVD occur in low- and middle-income countries [2]. Tobacco use, unhealthy diet, obesity, physical inactivity and harmful alcohol use have been recognized as the major behavioural CVD risk factors [2]. The presence of such modifiable CVD risk factors during emerging adulthood (stage between late adolescence and early adulthood) is a predecessor of CVDs in older adulthood [3, 4]. Emerging adulthood is an important period in health behaviour development as many of the health behaviours may change due to changing influences of parents, peers, social contexts and identity development [3, 5].

The largest increase in premature mortality attributable to CVD over the past 20 years was in East, South, and Southeast Asia; CVD accounted for 27% of all deaths in South Asia and 40% in East Asia in 2013 [6]. The prevalence of cardiovascular morbidity and its risk factors was found to be high in Southeast Asian countries [7], such as in Myanmar (9% angina and possible heart attack 7.5%) [8], Indonesia (smoking in men explained a 25% of coronary heart disease and 17% of strokes [9], and in Malaysia (among lifestyle risk factors: 72.8% inadequate vegetable and fruit intake and 41.3% physical inactivity [10]. More than 80% of the population consumes fewer than five servings of fruits and vegetables per day in five Association of Southeast Asian Nations (ASEAN) member states (Laos, Malaysia, Myanmar, Philippines, Vietnam) [7]. In addition, unhealthy dietary patterns include high fats and sugar intake as well as dietary salt [7]. Among the ten ASEAN countries (Brunei, Cambodia, Indonesia, Laos, Malaysia, Myanmar, Philippines, Singapore, Thailand, Vietnam) the smoking prevalence among men ranged from 23.1% in Singapore to 67.4% in Indonesia [11]. In Southeast Asia a high prevalence of problem drinking has been reported, e.g., 8.2% in the Thai population [12], 67% of men and 3% of women in Vietnam [13], and 25% in a rural community in Cambodia [14]. In the ASEAN region, the adult (> 20 years) prevalence of overweight (Body Mass Index = BMI ≥ 25–29.99 kg^2^) and obesity (BMI ≥ 30 kg^2^) ranged from in Vietnam (13.6% and 1.5%, respectively, among men, and 12.3% and 1.7%, respectively, among women) to in Malaysia (43.8% and 11.4% among men, and 48.6% and 16.7% among women) [15].

Knowledge about the link between behaviour and health (or risk awareness) is an important factor to inform choices about healthy behaviour [16]. An additional factor, closely linked to risk awareness, is health beliefs [16]. Understanding the prevalence of behavioural CVD risk factors, its risk awareness and health beliefs among emerging adults in ASEAN countries is of great importance for CVD prevention and control [17]. Little data are available on behavioural risk factors of CVD among emerging adults in ASEAN. Based on the Global School-based Health Survey (GSHS), 2007–2011, the prevalence of CVD risk factors in boys (13–15 years) was: tobacco use (Indonesia = 23.0%, Myanmar = 6.5%, Philippines = 7.0%, Thailand = 16.6%), alcohol use (Indonesia = 2.1%, Myanmar = 0.7%, Philippines = 16.6%, Thailand = 13.5%), low fruit and vegetables intake (Indonesia = 74.6%, Myanmar = 83.4%, Philippines = 73.5%, Thailand = 64.9%), low physical activity (Indonesia = 69.4%, Myanmar = 66.2%, Philippines = 82.9%, Thailand = 62.7%) [18], and 11.5% overweight and obesity across seven ASEAN countries [19].

In a study among emerging adults in eight European countries, more than 70% had not avoided eating fat and cholesterol, more than 30% had not engaged in any exercise in the past two weeks, more than 30% smoked cigarettes and more than 25% had more than one drink per day in the past two weeks [16]. Regarding the relationships between risk awareness and these behaviours mixed results were found, positive associations were found between health benefits of dietary fat avoidance, regular exercise, not smoking, not drinking too much alcohol consumption and respective health behaviours [16].

The aim of the study was to investigate the prevalence, risk awareness and health beliefs of behavioural risk factors (tobacco use, unhealthy diet, obesity, physical inactivity and harmful use of alcohol) for cardiovascular disease among university students in nine ASEAN countries (Cambodia, Indonesia, Laos, Malaysia, Myanmar, Philippines, Singapore, Thailand and Vietnam).

## Methods

### Sample and procedure

This was a cross-sectional study using a self-administered questionnaire on a range of health behaviours in university students in ASEAN countries. The university selection was a convenient sample. The questionaire used included standardized questionnaires that had been used previously [16, 20–22]. It was developed in English, then translated and back-translated into the languages (Bahasa, Khmer, Lao, Myanmar, Thai, and Vietnamese) of the participating countries. In every participating country, the questionnaire was pre-tested on a sample of 20–30 university students for validity; these results did not form part of the final sample. In each study country, undergraduate students were surveyed in classrooms selected through a cluster random sample procedure (one university department randomly selected from each faculty as a primary sampling unit, and for each selected department randomly ordered undergraduate courses). Informed consent was obtained from participating students, and the study was conducted in 2015. Participation rates were in Cambodia 93%, Indonesia 86%, Laos 90.8%, Malaysia 90.4%, Myanmar 73%, Singapore 74.4%, Thailand 97.3%, and Vietnam 92%. The sample size was calculated using Epi-Info Version 7.1 (Centers for Disease Control and Prevention, Atlanta, GA; USA). For the population survey, the minimum sample size at confidence level of 99% was calculated as 663 per country.

### Measures

*Socio-demographic* questions included age, gender, and subjective socioeconomic background [16].

#### Behavioural risk factors for CVD

*Unhealthy diet* was assessed with the question, “Do you make a conscious effort to avoid eating foods that contain fat and cholesterol?” [16].

#### Anthropometric measurements

Students were weighed and height measured by trained researchers using standardised procedures [23], except for in Cambodia where self-reported assessment was used. BMI was classified according to Asian criteria: normal weight (18.5 to < 23.0 kg/m^2^), overweight (23.0 to < 25.0 kg/m^2^) and 25.0 or more kg/m^2^ as obese [24].

*Tobacco use* was assessed with the question: “Do you currently use one or more of the following tobacco products (cigarettes, snuff, chewing tobacco, cigars, etc.)?” Response options were “yes” or “no” [20].

*Past month binge drinking* was assessed with one item of the “Alcohol Use Disorder Identification Test” [21].

*Physical activity* was assessed using the six items in the “International Physical Activity Questionnaire” (IPAQ) short form questionnaire. We used the instructions given in the IPAQ manual and calculated three levels of physical acidity (low, moderate and high) [22].

#### Risk awareness

The risk awareness items included the knowledge (yes/no) whether or not each of the health behaviours contributed to health problems. For *overweight*, heart disease and high blood pressure were acceptable (0–2), for *exercise,* heart disease and high blood pressure (0–2), for *smoking*, heart disease, lung cancer and high blood pressure (0–3), for *alcohol,* heart disease, high blood pressure (0–2), and for *eating fat,* heart disease and breast cancer (0–2) [16, 25].

#### Beliefs in health benefits

Study participants were asked to rate the importance of five health behaviours (keep body weight within normal range, take regular exercise, non-smoking, not drinking too much alcohol, and not to eat too much animal fat) for health maintenance on 10-point scales, ranging from 1 = of very low importance to 10 = of very great importance [16].

*Self-rated health status* was assessed by a single item, “In general, would you say that your health is … excellent, very good, good, fair or poor” [26].

### Data analysis

Data analysis was performed using STATA software version 13.0 (Stata Corporation, College Station, Texas, USA). Descriptive statistics were used to calculate frequency of sample characteristics of the study population. Differences in proportions were tested by Mann-Whitney U Tests. Multivariable logistic regression analysis was performed with each behavioural CVD risk factor (overweight or obesity, low physical activity, tobacco use, binge drinking and not avoiding fat and cholesterol) as the dependent variable. Socio-demographic characteristics, subjective health status, risk awareness and health benefits variables were taken as independent variables. *P* < 0.05 was considered significant. Since the study used a clustered design, country was included as a clustering variable in the regression models.

## Results

The sample included 8806 (37.5% male and 62.5% female) university students (Mean age 20.6, SD = 2.0) from nine ASEAN countries. The sample size differed by country, ranging from 491 in Myanmar to 1658 in Thailand (see Table 1).

**Table 1:** Sample characteristics

Country	Total number	Men	Women	Age	Tertiary enrolment ratio, 2013–2015 [31]
	N (%)	n (%)	n (%)	Mean (SD)	
Cambodia^a^	1357 (15.4)	667 (20.2)	690 (12.5)	21.3 (2.2)	13.1
Indonesia^a^	981 (11.1)	286 (8.7)	695 (12.6)	19.3 (2.4)	24.3
Laos^a^	806 (9.1)	273 (8.3)	533 (9.7)	22.2 (1.8)	16.9
Malaysia^b^	1023 (11.6)	504 (15.3)	519 (9.4)	20.7 (1.4)	26.1
Myanmar^a^	491 (5.6)	209 (6.3)	282 (5.1)	20.1 (1.1)	13.5
Philippines^a^	782 (8.9)	201 (6.1)	581 (10.6)	18.7 (1.0)	35.7
Singapore^c^	891 (10.1)	449 (13.6)	442 (8.0)	21.2 (1.6)	69.8
Thailand^b^	1658 (18.8)	308 (9.3)	1350 (24.5)	20.0 (1.3)	48.9
Vietnam^a^	817 (9.3)	404 (12.2)	413 (7.5)	21.4 (1.6)	28.8
All	8806	3301 (37.5)	5505 (62.5)	20.6 (2.0)	

^a^lower middle income country; ^b^upper middle income country; ^c^high income country [32]

### Prevalence of behavioural risk factors

The prevalence of five CVD behavioural risks (overweight, physical activity, tobacco use, binge drinking and dietary behaviour) among men and women from each country is described in Table 2. Across all nine countries, among men 27.5% were overweight or obese, 39.0% engaged in low physical activity, 6.9% were current tobacco users, 10.1% had engaged in binge drinking in the past month and 62.7% did not avoid eating fat and cholesterol, while among women 16.9% were overweight or obese, 53.0% engaged in low physical activity, 2.5% had used tobacco in the past month, 4.2% had engaged in binge drinking in the past month and 58.2% did not avoid eating fat and cholesterol. There were country differences regarding all five behavioural risk factors for men and for women. For instance, among men, overweight or obesity was the highest in Myanmar (38.6%) and Malaysia (38.2%) and the lowest in Cambodia (15.2%), and among women overweight or obesity was the highest in Malaysia (27.0%) and the lowest in Vietnam (6.2%). Physical inactivity among men was the highest in Cambodia (56.1%) and the lowest in Laos (26.0%), and among women it was the highest in Cambodia (65.1%) as well and the lowest in Vietnam (34.1%). The current tobacco use prevalence was below 2% among men in Cambodia and among women in Cambodia, Malaysia, Myanmar, Singapore and Vietnam. Binge drinking was the highest among men and women in Laos (46.2% and 13.3%, respectively) and the lowest below 1.5% among men in Indonesia and Malaysia and among women in Cambodia, Indonesia, Malaysia, Myanmar and Vietnam. The non-avoidance of fat and cholesterol was, among men, above 70% in Laos and Vietnam, and above 65% in Malaysia, Philippines and Singapore, while, among women, it was 50% in Singapore and Thailand and around 70% in Laos and the Philippines (see Table 2).

**Table 2:** Prevalence of behavioural CVD risk factors among men and women in nine ASEAN countries

Country	Body mass index-Overweight or obesity	Physical inactivity	Tobacco use	Binge drinking	Non-avoidance of fat and cholesterol
	%	%	%	%	%
Men					
Cambodia	15.2 (12.7, 18.1)	56.1 (52.4, 59.8)	1.3 (0.7, 2.5)	7.5 (5.8, 9.8)	Not assessed
Indonesia	38.2 (32.7, 44.0)	48.4 (42.6, 54.3)	18.9 (14.7, 23.8)	1.4 (0.5, 3.7)	50.5 (44.7, 56.4)
Laos	23.8 (19.0, 29.4)	26.0 (21.1, 31.5)	10.6 (7.5, 14.9)	46.2 (40.3, 52.1)	72.2 (66.5, 77.2)
Malaysia	38.2 (34.0, 42.5)	30.0 (26.1, 34.1)	6.0 (4.2, 8.4)	0.2 (0.03, 1.4)	66.1 (61.8, 70.1)
Myanmar	38.6 (30.7, 47.2)	44.9 (39.8, 49.8)	2.4 (1.0, 5.6)	3.3 (1.6, 6.9)	51.0 (44.1, 57.8)
Philippines	34.0 (27.7, 41.0)	33.0 (26.9, 39.7)	13.6 (9.5, 19.1)	13.4 (9.4, 18.9)	68.0 (61.2, 74.1)
Singapore	29.3 (24.6, 34.4)	31.4 (27.2, 35.9)	5.8 (4.0, 8.4)	8.5(6.2, 11.4)	66.2 (57.6, 66.6)
Thailand	25.5 (20.8, 30.8)	42.8 (37.2, 48.5)	12.3 (9.0, 16.6)	22.4 (18.1, 27.4)	53.1 (47.3, 58.7)
Vietnam	22.3 (18.3, 26.9)	32.4 (28.0, 37.2)	3.7 (2.1, 6.1)	2.7 (1.5, 4.9)	71.5 (66.9, 75.7)
All	27.5 (26.0, 29.2)	39.0 (37.3, 40.7)	6.9 (6.2, 7.9)	10.1 (9.1, 11.1)	62.7 (60.8, 64.5)
Women					
Cambodia	9.3 (7.3, 11.7)	65.1 (61.3, 68.6)	0.9 (0.4, 2.0)	0.4 (0.1, 1.4)	Not assessed
Indonesia	18.7 (15.9, 21.8)	59.4 (55.7, 63.1)	3.6 (2.4, 5.3)	0.4 (0.1, 1.3)	55.0 (51.3, 58.7)
Laos	18.8 (15.6, 22.5)	62.1 (57.9, 66.1)	6.2 (4.4, 8.6)	13.3 (10.7, 16.5)	69.6 (65.6, 73.4)
Malaysia	27.0 (23.3, 30.9)	49.5 (45.2, 53.8)	0.2 (0.03, 1.4)	0.4 (0.1, 1.5)	58.7 (54.4, 62.9)
Myanmar	22.9 (17.6, 29.2)	58.9 (55.8, 62.7)	0.0	0.0	61.3 (55.4, 66.8)
Philippines	18.6 (15.6, 22.0)	45.2 (41.2, 49.3)	4.0 (2.7, 5.9)	3.8 (2.5, 5.7)	72.6 (68.9, 76.1)
Singapore	15.6 (12.1, 19.8)	46.4 (41.7, 51.0)	1.1 (0.5, 2.7)	4.3 (2.8, 6.6)	50.1 (45.5, 54.8)
Thailand	17.2 (15.3, 19.4)	52.8 (50.1, 55.6)	2.8 (2.0, 3.9)	8.1 (6.8, 9.7)	49.9 (47.2, 52.6)
Vietnam	6.2 (4.2, 9.0)	34.1 (29.7, 38.9)	0.2 (0.03, 1.7)	0.2 (0.03, 1.7)	61.2 (56.4, 65.8)
All	16.9 (15.9, 17.9)	53.0 (51.6, 54.4)	2.5 (2.0, 2.8)	4.2 (3.7, 4.8)	58.2 (56.8, 59.6)

#### Risk awareness

The lowest risk awareness was found from the link between exercise, heart disease and high blood pressure (0.78, range 0–2) and eating fat, heart disease and breast cancer (0.83, range 0–2), followed by alcohol use links (1.14, range 0–2) and overweight links (1.46, range 0–2). For the link between smoking heart disease, lung cancer and high blood pressure the risk awareness score was 1.77 (range 0–3). Generally, men had a higher risk awareness score than women, in particular for overweight, cigarette smoking and dietary fat. There were hardly any country differences, and also not country gender differences regarding the different health risk behaviour awareness ratings (see Table 3).

**Table 3:** Health risk behaviour awareness ratings (Means)

Country^a^	Overweight (0–2)			Lack of exercise (0–2)			Cigarette smoking (0–3)			Alcohol use (0–2)			Dietary fat (0–2)		
	All	Men	Women	All	Men	Women	All	Men	Women	All	Men	Women	All	Men	Women
Indonesia	1.30	1.22	1.33	0.58	0.50	0.61	2.07	1.87	2.15*	1.19	1.09	1.23	0.88	0.82	0.98*
Laos	1.08	1.02	1.11	0.52	0.57	0.50	1.34	1.33	1.35	0.74	0.67	0.78	0.62	0.66	0.60
Malaysia	1.52	1.55	1.49	0.65	0.64	0.65	1.75	1.81	1.69	1.04	1.04	1.03	0.89	0.88	0.90
Myanmar	1.68	1.65	1.71	0.88	0.89	0.88	2.10	2.28	1.98	1.14	1.19	1.10	0.75	1.12*	0.59
Philippines	1.73	1.81	1.70	0.73	0.76	0.72	1.78	1.90	1.74	1.27	1.39	1.23	0.98	1.09	0.96
Singapore	1.68	1.68	1.68	0.91	0.90	0.93	2.02	2.04	2.00	1.28	1.24	1.31	0.93	0.92	0.93
Thailand	1.28	1.27	1.29	0.69	0.63	0.70	1.43	1.56	1.40	1.09	1.15	1.08	0.73	0.72	0.74
Vietnam	1.72	1.73	1.72	1.32	1.24	1.39	2.14	2.21	2.07	1.38	1.41	1.35	1.06	1.05	1.06
All	1.46	1.50*	1.44	0.78	0.80	0.76	1.77	1.87*	1.72	1.14	1.15	1.13	0.83	0.85*	0.82

*P < 0.001; ^a^was not assessed in Cambodia

#### Health benefits ratings

Overall, the importance of not smoking and not drinking too much alcohol was endorsed with the highest ratings, while the lowest ratings were given for not eating too much fat. Except for taking regular exercise, women endorsed each health behaviour (keeping body weight within normal range, not smoking, not drinking too much and not eating too much fat) as more important than men. Among the different countries, keeping body weight within normal range was rated with the highest importance in Indonesia (8.5, range 1–10). Taking regular exercise was, among men, given the highest importance in Laos (8.4), and among women in Cambodia (8.2), while the lowest importance of taking regular exercise was found among Thai students. The highest endorsement for not smoking was found among students in the Philippines (9.5) and the lowest importance was found in Laos (7.3). Not drinking too much alcohol was highly endorsed by students from all countries, and was the highest, among men, in Cambodia (9.1) and among women in Malaysia. The highest ratings for not eating too much fat were in Indonesia (7.5) and Vietnam (7.4), while the lowest importance of not eating too much fat was in Laos (5.7) (see Table 4).

**Table 4:** Health behaviour benefits ratings (1–10 scale)

Country	Keep body weight within normal range			Taking regular exercise			Not smoking			Not drinking too much alcohol			Not eating too much fat		
	All	Men	Women	All	Men	Women	All	Men	Women	All	Men	Women	All	Men	Women
Cambodia	8.05	8.15	7.94	8.17	8.09	8.24	8.49	8.52	8.47	8.98	9.05	8.92	6.27	6.44	6.10
Indonesia	8.53	8.40	8.58	7.97	8.19	7.88	8.91	8.30	9.16*	9.09	8.83	9.20*	7.54	7.45	7.58
Laos	7.86	7.66	7.96	7.84	8.40*	7.56	7.25	6.42	7.68*	7.88	7.40	8.12*	5.69	5.16	5.96*
Malaysia	8.25	8.05	8.44*	7.91	8.12	7.71	9.22	8.99	9.44*	8.94	8.58	9.29*	7.22	7.01	7.43*
Myanmar	7.50	7.25	7.67	7.13	7.22	7.06	8.76	8.16	9.20*	8.43	8.18	8.61	6.98	6.68	7.20
Philippines	8.31	7.98	8.43	7.26	7.53	7.16	9.50	9.15	9.62*	8.84	8.12	9.09*	6.64	6.46	6.70
Singapore	7.78	7.57	7.99	8.01	8.22*	7.80	8.87	8.72	9.01	8.01	7.74	8.28*	6.79	6.59	6.98
Thailand	7.51	7.23	7.57	6.40	6.88*	6.29	8.63	8.17	8.74*	8.36	7.64	8.52*	6.29	6.40	6.27
Vietnam	7.96	7.61	8.30*	7.93	7.92	7.93	9.15	8.92*	9.38	8.57	8.26	8.87*	7.35	7.02	7.68*
All	7.96	7.82	8.05*	7.58	7.92*	7.37	8.74	8.46	8.91*	8.59	8.30	8.76*	6.67	6.59	6.71

*P < 0.001

### Predictors of behavioural CVD risk factors

Multivariable logistic regressions were conducted on overweight, low physical activity, tobacco use, binge drinking and dietary behaviour (not avoiding eating fat and cholesterol). Regarding overweight or obesity, being male (OR = 1.68, CI = 1.49–1.90), coming from a wealthy family background (OR = 1.26, CI = 1.10–1.43), and lack of benefits beliefs on the importance of keeping the body weight within normal range (OR = 0.97, CI = 0.94–0.97) was associated with being overweight, while no association was found between risk awareness of overweight and being overweight. In terms of low physical activity, being female (OR = 0.57, CI = 0.51–0.63), poor subjective health status (OR = 1.12, CI = 1.06–1.18), and low benefits beliefs on the importance of taking regular exercise (OR = 0.90, CI = 0.89–0.92) was associated with low physical activity, while no association was found between risk awareness of exercise and low physical activity. In relation to tobacco use and binge drinking, being male (OR = 3.17, CI = 2.49–4.03 and OR = 2.18, CI = 1.80–2.64, respectively), older age (OR = 1.34, CI = 1.00–1.80 and OR = 2.22, CI = 1.72–2.88), lower risk awareness (OR = 0.88, CI = 0.77–1.00 and OR = 0.79, CI = 0.70–0.90), and lower health benefits beliefs (OR = 0.79, CI = 0.77–0.81 and OR = 0.83, CI = 80–0.85, respectively), were both associated with tobacco use and binge drinking. In addition, having a wealthy family background (OR = 1.69, CI = 1.31–2.17), and coming from a lower middle income country (OR = 1.55, CI = 1.20–2.00) was associated with tobacco use, and having a poorer subjective health status (OR = 1.17, CI = 1.05–1.27) was associated with binge drinking. In terms of dietary behaviour, being male (OR = 1.20, CI = 1.08–1.33), older age (22–30 years) (OR = 1.15, CI = 1.01–1.32), living in a lower middle income country (OR = 1.56, CI = 1.40–1.74), poor subjective health status (OR = 1.08, CI = 1.02–1.14), and low health beliefs in avoiding fat and cholesterol (OR = 0.84, CI = 0.82–0.85) was associated with non-avoidance of fat and cholesterol (see Table 5).

**Table 5:** Predictors of behavioural CVD risk factors

Variable	Overweight or obesity vs. normal weight	Low physical activity	Tobacco use	Binge drinking	Not avoiding fat and cholesterol
	AOR (95% CI)	AOR (95% CI)	AOR (95% CI)	AOR (95% CI)	AOR (95% CI)
Gender					
Female	1 (Reference)	1 (Reference)	1 (Reference)	1 (Reference)	1 (Reference)
Male	1.68 (1.49–1.90)***	0.57 (0.51–0.63)***	3.17 (2.49–4.03)***	2.18 (1.80–2.64)***	1.20 (1.08–1.33)***
Age					
18–20	1 (Reference)	1 (Reference)	1 (Reference)	1 (Reference)	1 (Reference)
20–21	0.90 (0.78–1.05)	1.02 (0.91–1.15)	0.72 (0.53–0.98)*	1.52 (1.18–1.97)***	0.89 (0.79–1.00)*
22–30	1.07 (0.92–1.26)	1.01 (0.89–1.15)	1.34 (1.00–1.80*	2.22 1.72–2.88)***	1.15 (1.01–1.32)*
Family wealth					
Poor	1 (Reference)	1 (Reference)	1 (Reference)	1 (Reference)	1 (Reference)
Wealthy	1.26 (1.10–1.43)***	1.22 (1.10–1.36)	1.69 (1.31–2.17)***	1.03 (0.83–1.27)	1.04 (0.93–1.15)
Country income					
Upper middle/high	1 (Reference)	1 (Reference)	1 (Reference)	1 (Reference)	1 (Reference)
Lower middle	0.91 (0.79–1.03)	1.03 (0.93–1.15)	1.55 (1.20–2.00)**	1.07 (0.87–1.31)	1.56 (1.40–1.74)***
Residence					
Away from parents	1 (Reference)	1 (Reference)	1 (Reference)	1 (Reference)	1 (Reference)
With parents	0.99 (0.86–1.13)	0.94 (0.84–1.05)	1.12 (0.87–1.43)	0.91 (0.76–1.12)	1.00 (0.90–1.12)
Negative subjective health					
status (scale)	0.94 (0.87–1.00	1.12 (1.06–1.18)***	0.97 (0.86–1.09)	1.17 (1.05–1.27)*	1.08 (1.02–1.14)**
Risk awareness (scale)	0.99 (0.89–1.09)a	0.97 (0.91–1.03)b	0.88 (0.77–1.00)*c	0.79 (0.70–0.90)***d	1.03 (0.95–1.11)e
Benefits (scale)	0.97 (0.94–0.97)*A	0.90 (0.89–0.92)***B	0.79 (0.77–0.81)***C	0.83 (0.80–0.85)***D	0.84 (0.82–0.85)***E

*AOR* Adjusted Odds Ratio, *CI* Confidence Interval, ***P < 0.001, **P < 0.01, * < 0.05

*a* being overweight, *b* exercise, *c* smoking, *d* alcohol, *e* eating fat

*A* keep body weight within normal range, *B* take regular exercise, *C* non-smoking, *D* not drinking too much alcohol, *E* not to eat too much animal fat

## Discussion

This study investigated male and female university students’ CVD behavioural risk factors, its risk awareness and health benefits in a large sample of university students in nine ASEAN countries. The prevalence of behavioural CVD risk factors (overweight, low physical activity, tobacco use, binge drinking, and avoidance of eating fat) in this study was similar to a study among emerging adults in eight European countries, except for tobacco use, which was lower in this study than among European university students [16]. Regarding obesity, this study found a higher prevalence than among male school-going adolescents across seven ASEAN countries (11.5%) [19], while in relation to the prevalence of low physical activity and current tobacco use, this study found a lower prevalence than among male school-going adolescents across the ASEAN countries (70% and 13%, respectively) [18]. Considerable country variation was found across countries in all five behavioural risk factors, while these were similar for men and women. Men had a higher prevalence of behavioural risk factors than women in terms of obesity, tobacco use, binge drinking and eating foods containing fat and cholesterol, while women had a higher prevalence of low physical activity than men. Similar gender differences were found in previous studies [e.g., 16]. Compared to the other study countries, Cambodia had the lowest prevalence of overweight or obesity (12.2%). One possible reason for this could be that in this study body weight and height were assessed by self-report in Cambodia and in the other countries BMI was measured. It is possible that the BMI in Cambodia based on self-report under-reported BMI [27]. Previous national studies in Cambodia found among adolescents, also by self-report, 3.7% [19] and among 25 to 34 year-olds, based on measured BMI, 23.4% overweight or obesity [28], supporting a relatively low prevalence of overweight or obesity in Cambodia.

Risk awareness was assessed with the endorsement of relationships between five behavioural CVD risk factors and a range of specific illnesses. Generally, the risk awareness for the five behavioural CVD risk factors was low, but comparable to what was found in a European emerging adults study, e.g. 0.78 (range 0–2) for exercise, compared to 0.86 in the European study, cigarette smoking 1.79 (range 0–3), compared to 1.92, alcohol use 1.14 (range 0–2), compared to 1.11, and dietary fat 0.82 (range 0–2), compared to 0.78 in the European study [16]. Generally, men had higher risk awareness scores than women, meaning an increased need to promote more risk awareness among female university students.

The assessment of health benefits of five health behaviours found that university students seem to be given high ratings of each health behaviour in their importance to health, which was also found in a previous study [16]. Not smoking (8.7, range 1–10) and not drinking too much (8.5) were rated overall as more important than keeping the body weight within normal range (7.9), which was in turn rated more important than taking regular exercises (7.6) and not eating too much fat (6.7). As found in a previous study [16], in this study, women reported a ‘healthier’ belief profile than men except in the domain of exercise, which female students rated as less important than men.

In multivariable logistic regression, lack of risk awareness was found to be associated with tobacco use and binge drinking, however, no associations were found between risk awareness and overweight, low physical activity and dietary behaviour (non-avoidance of fat and cholesterol). One message from this finding could be that risk awareness programmes targeting tobacco use and alcohol drinking could be effective, while such programs may not be effective for the other behavioural risk factors (overweight, low physical activity and poor dietary behaviour). Other studies (e.g., [16]) have also found that the relationship between risk awareness and health behaviour is not straight forward.

After adjusting for socio-demographic factors, health status and risk awareness, poorer health benefits beliefs predicted overweight, low physical activity, tobacco use, binge drinking and non-avoidance of fat and cholesterol. These findings are in agreement with previous studies [16, 29] showing that beliefs in the value of a preventive action are associated with the probability that the behaviour is performed.

Because of the effect on productivity and health of the population, the epidemic of cardiovascular behavioural risk factors in ASEAN needs urgent attention [7] Although there is an emergence of national policies and non-communicable Disease, including CVD, control programmes in the ASEAN region, efforts need to be considerably strengthened [7]. Interventions targeting this university student population should improve dietary behaviour, decrease alcohol use, stop tobacco use and increase physical activity by using “multiple health behaviour change interventions” that could be administered through university health centres and healt promotion programmes [3]. Further, previously shown effective [30], structured educational programmes promoting a healthy lifestyle can be incorporated to the university curriculum.

### Study limitations

This study had several limitations. The study was cross-sectional, so causal conclusions cannot be drawn. The investigation was carried out with students from one or two universities in each country, and inclusion of other centres could have resulted in different results. University students are not representative of young adults in general, since in most study countries only a minority enrol in tertiary education (13.1–35.7%), with only higher rates of gross enrolment ratios in Thailand (48.9%) and Singapore (69.8%). Consequently, behavioural risk factors and its beliefs and attitudes may be different in other sectors of the population.

## Conclusion

The study found among a large sample of ASEAN university students a high prevalence of behavioural risk factors of CVD, and risk awareness was partially and positive health beliefs were negatively associated with behavioural risk factors. Since main CVD risk factors, including overweight, unhealthy diet, physical inactivity, tobacco use and binge drinking, were prevalent in this emerging adult population the development of health promotion programmes targeting these risk factors in the university environment may help to prevent subsequent CVD development.

## Abbreviations

ASEAN: Association of Southeast Asian Nations
BMI: Body Mass Index
CVD: Cardiovascular disease
IPAQ: International Physical Activity Questionnaire
